# HIV testing frequency and associated factors among five key populations in ten cities of China: a cross-sectional study

**DOI:** 10.1186/s12879-022-07189-6

**Published:** 2022-02-28

**Authors:** Kedi Jiao, Ran Wei, Haochu Li, Eric P. F. Chow, Eduardo Piqueiras, Taylor Lewis, Zece Xu, Ci Ren, Wei Ma

**Affiliations:** 1 Department of Epidemiology, School of Public Health, Cheeloo College of Medicine, Shandong University, 44 West Wenhua Road, Jinan, Shandong 250012 People’s Republic of China; 2 Shandong Provincial Chest Hospital, Jinan, Shandong People’s Republic of China; 3 Melbourne Sexual Health Centre, Alfred Health, Carlton, Australia; 4 Central Clinical School, Monash University, Melbourne, VIC Australia; 5 Centre for Epidemiology and Biostatistics, Melbourne School of Population and Global Health, The University of Melbourne, Carlton, Australia; 6 Department of Anthropology, School of Social Work, Wayne State University, Detroit, MI USA; 7 Research Triangle Institute (RTI) International, Washington, DC USA

**Keywords:** HIV/AIDS, HIV testing, Associated factors, Key populations, China

## Abstract

**Background:**

Regular HIV testing is the best way to detect people living with HIV promptly, yet not much is known about the characteristics of frequent, voluntary testers. This study explores factors related to HIV testing frequency among five key populations in China including men who have sex with men (MSM), female sex workers (FSWs), people who use drugs (PWUD), men who have casual sex with women (MCSW) and sero-negative partners among sero-discordant couples (SNPs).

**Methods:**

We conducted a cross-sectional study in ten cities of China from November 2018 to September 2019 using convenience sampling to recruit participants. Univariate and multivariate partial proportional odds models were adopted to compare socio-behavioral factors associated with HIV testing frequencies among the five key populations.

**Results:**

Among the 2022 recruited participants, 36.6% reported not testing for HIV in the past year, whereas 37.0% tested once and 26.4% tested twice. Compared with MSM, FSWs (AOR = 1.97, 95% CI: 1.36–2.86) and SNPs (AOR = 3.63, 95% CI: 2.40–5.49) were more likely to test for HIV, but MCSW (AOR = 0.23, 95% CI: 0.17–0.32) were less likely. Additionally, SNPs (AOR = 4.02, 95% CI: 2.78–5.83) were more likely to be frequent HIV testers, while FSWs (AOR = 0.49, 95% CI: 0.32–0.76) and MCSW (AOR = 0.29, 95% CI: 0.20–0.41) were less likely to be frequent testers. Factors identified as barriers to HIV testing include the following: higher education level and > 5000 CNY monthly income for FSWs; elder age and a married/cohabitating status for PWUD; reported alcohol use for MCSW; and non-Han ethnicity and non-local household for SNPs. Facilitators to frequent testing included the following: higher education level for MSM and SNPs; higher AIDS knowledge score for MSM and PWUD; > 5000 CNY monthly income for FSWs and PWUD; and reporting high-risk sexual behaviors for MSM, FSW and PWUD.

**Conclusions:**

HIV testing frequencies and associated factors were not equivalent across the five key populations in China. Public health officials should take heed of the identified high-risk populations reporting high testing rates, perhaps with intensive and tailored behavioral interventions or biochemical prophylaxis.

**Supplementary Information:**

The online version contains supplementary material available at 10.1186/s12879-022-07189-6.

## Background

As of October 2019, China’s National Health Commission has reported an estimated 958,000 people living with HIV who have been tested and thus know their sero-status [1]. Increasing HIV testing among at-risk individuals and linking HIV-positive individuals to care has been identified as a critical strategy to mitigate the ongoing transmission of HIV [2, 3]. The WHO recommends that key populations, including men who have sex with men (MSM), persons who exchange sex for money, and drug users, should be tested for HIV at least once a year [4].

Regular HIV testing is the gateway to detecting people living with HIV promptly, but more research is needed to learn about the risk-profile and characteristics of frequent testers. Studies on the facilitators and barriers to HIV testing among different key populations have found links with socio-demographic factors (e.g., age, education level, marital status) as well as behavioral factors (e.g., multiple sexual partners, unprotected sex, drug use)[5–8]. However, there is a dearth of literature about factors associated with frequent HIV testing among key populations other than MSM. In and of itself, high-risk sexual behavior is considered a key correlate of frequent HIV testing, yet its effect is inconsistent [9–12]. For instance, studies in London and Germany have found that frequent HIV testing is associated with reported unprotected anal sex or condomless sex with casual partners [9, 11]. But a study in Peru indicated that those reporting unprotected anal sex were less likely to test frequently [10].

In China, the HIV epidemic is mainly concentrated among MSM, female sex workers (FSWs), people who use drugs (PWUD) and male patients of sexually transmitted disease clinics [13]. These are considered high-risk groups and are included in Chinese HIV Sentinel Surveillance System [14]. In addition, data shows that about 25% of heterosexual HIV transmission is through sexual contact with one’s partner, indicating the transmission between sero-discordant couples plays a non-trivial role of new infections [15]. Hence, one of the key populations included in this study is sero-negative partners among sero-discordant couples, or SNPs.

Current policies in China focus on expanding coverage of testing without offering specific guidelines on recommended HIV testing frequency. This is potentially problematic because previous studies have suggested HIV testing uptake is insufficient among certain key populations such as PWUD (25.8% in 2018) [8] and male clients of FSWs (23.8% in 2019) [16]. With respect to MSM, a study in Beijing showed 71% reported ever testing for HIV and 52% reported ≥ 2 HIV testing in their lifetimes [17]. Another study of MSM in Guangzhou defining frequent testers as those who did so at least twice per year found that 44.3% were tested frequently [18]. Some of psychosocial factors associated with frequent testing were having disclosed sexual orientation to non-gay friends and having lower internalized homophobia [18].

There have been few studies exploring the socio-behavioral factors associated with HIV testing frequency, especially in China. Again, the literature focuses primarily on MSM, ignoring other key populations at risk of HIV acquisition. It remains unclear whether the socio-behavioral factors impacting HIV testing frequency are consistent among these other key populations. This study aims to fill that research gap by examining and comparing HIV testing frequency and associated factors among five key populations in China that includes not only MSM, but also FSWs, PWUD, SNPs, and men who have casual sex with women (MCSW). Findings from this article could help public officials improve practice and potentially re-allocate resources to decrease the risk of further HIV transmission among these high-risk subgroups.

## Methods

### Study sites and study samples

**Fig. 1:**
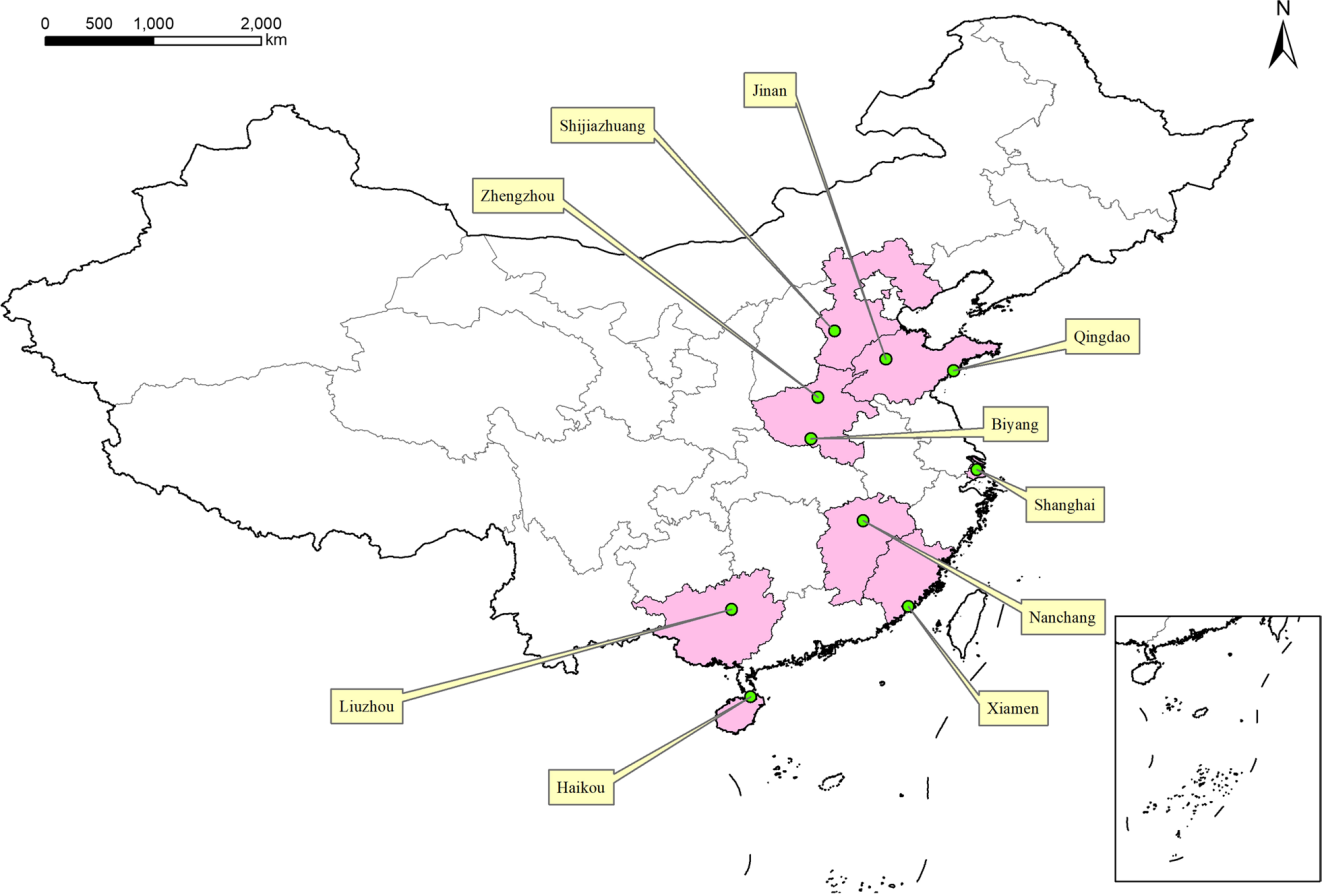
Location of the ten study sites (Shijiazhuang, Xiamen, Zhengzhou, Nanchang, Biyang, Qingdao, Shanghai, Jinan, Haikou, and Liuzhou)

A cross-sectional study was conducted in ten cities in China from November 2018 to September 2019. Based on the literature and recommendations by experts from the China’s Centers for Disease Control and Prevention (CDC), participants were recruited as follows: MSM from Shijiazhuang and Xiamen [19], FSWs from Zhengzhou, Nanchang and Biyang [20], PWUD from Qingdao and Shanghai [8, 21], MCSW from Jinan and Haikou[13], and SNPs from Zhengzhou and Liuzhou [15, 22]. (Fig. 1).

Eligibility criteria of participants in this study were (1) age 18 years or older; (2) self-reported HIV status of negative or unknown; (3) providing informed consent to participate in the study; and (4) exhibiting one the following behavioral characteristics: male who had anal sex with men in the past 12 months, for MSM; female who provided commercial sex for money or goods in the past 12 months, for FSWs; taking illicit drugs in the past 12 months, for PWUD; male who had casual sex with female in the past 12 months, for MCSW, including those who had casual sex through commercial ways (i.e. male clients of FSW) or those who had casual sex through non-marital and non-commercial temporary ways [23]; people whose spouse had been diagnosed as HIV-positive, for SNPs. Based on assumed HIV testing rates of the five populations, we targeted a sample size of 400 participants for each key population and about 2000 for all five populations (see Additional file 1 for details).

### Recruitment of participants

We used convenience sampling to recruit participants through referrals from local community-based organizations (CBOs), CDCs, and hospitals. For MSM, FSWs and PWUD, outreach workers from local CBOs approached potential participants in entertainment venues and workplaces of FSWs (e.g., hair salons, saunas, bath centers, karaoke bars, dancing hall, hotels, nightclubs, massage parlors, guesthouses). For MCSW, practitioners from local hospitals identified and approached potential participants among male clients of sexually transmitted diseases clinics. For SNPs, workers of local CDCs/hospitals identified potential participants from medical records and contacted them. During the recruitment process, trained investigators first verified each participant’s eligibility, and then fully explained the purpose, contents, and procedures of the study to them. Conditional on providing written informed consent, each participant was asked to complete a self-administrated questionnaire. The participants were assured their data would be kept confidential and only reported in a summarized format. After finishing the survey, each participant was reimbursed 50 CNY (about 7.07 USD) for their time.

### Measures and variables

Five structured questionnaires, one for each of the key populations, were developed based on China’s National Sentinel Surveillance questionnaire and a complementary review of the literature. We collected socio-demographic information (including age, ethnicity, whether from a local household and for how long living there, education level, and monthly income, marital status), AIDS knowledge, and behavioral information (including alcohol consumption and specific high-risk sexual behaviors tailored for each key population).

We defined “HIV testing frequency” via literature review [18] and from China CDC recommendations. Specifically, the four outcome variables were identified from the question “How often did you take an HIV test in the past 12 months?” with response options “never”, “once” and “twice or above”. We define “no testing” as individuals who had not tested for HIV in the past 12 months, “ever testing” as individuals who had tested for HIV at least once in the past 12 months, “infrequent testing” as individuals who had tested for HIV once or less in the past 12 months, and “frequent testing” as individuals who had tested for HIV twice or more in the past 12 months.

### Statistical analysis

Descriptive analyses consisting of counts and proportions were used to summarize the socio-demographic and behavioral characteristics. A partial proportional odds model (PPOM) was adopted to account for ordinal responses [24]. The traditional ordinal logistic model assumes that coefficients do not vary across cut point equations. The PPOM relaxes that assumption, allowing one to estimate additional coefficients for the independent variables violating the assumption. We assessed the proportional odds assumption using score tests (see Table S1-S6 of Additional file 2). Any independent variable violating the assumption was permitted to have unequal slopes in relation to the dependent variable.

We first fitted univariate and multivariate PPOMs for all five populations pooled together in order to compare HIV testing frequency amongst the populations. We included only the socio-demographic characteristics in common for all five populations (see Table 1 in Results section). Next, we conducted stratified analyses and fitted univariate and multivariate PPOMs for each key population respectively to explore factors associated with HIV testing frequency. In the population-specific PPOMs, we included not only socio-demographic characteristics in common for all five populations (see Table 1 in Results section), but also high-risk behaviors for each population (see Table 2 in Results section). Independent variables at a significance level of P < 0.1 in univariate analyses were included in the multivariate PPOMs. Both crude odds ratio (OR) and adjusted odds ratio (AOR) were calculated and presented with 95% confidence intervals (CIs) and P-values.

All data management and descriptive analyses were conducted using SPSS24.0, but the univariate and multivariate PPOMs were fitted using SAS9.4.

## Results

### Socio-demographic and behavioral characteristics of the five key populations

**Fig. 2:**
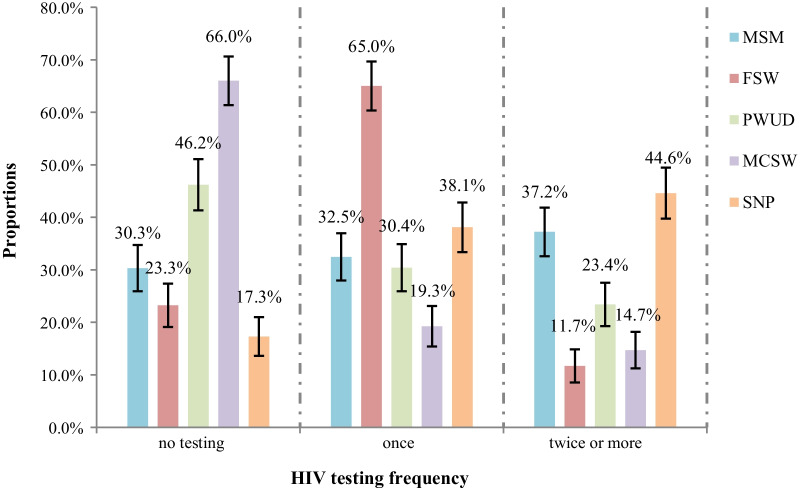
Proportions of HIV testing frequency in the past 12 months among five key populations in China. Total N = 2022, 737 (36.4%) did not take an HIV testing, 746 (36.9%) tested only once, and 533 (26.4%) took two or more testing in the past year. There are 3 missing values of HIV testing frequency for PWUD and SNPs respectively. The error bars represent the 95% confidence intervals. *MSM* men who have sex with men, *FSW* female sex worker, *PWUD* people who use drugs; *MCSW* men who have casual sex with women, *SNP* sero-negative partner among sero-discordant couples

A total of 2022 eligible participants were included in our analysis, consisting of 419 MSM, 400 FSWs, 401 PWUD, 400 MCSW and 402 SNPs. Of the 2022 participants, 737 (36.6%) had not taking an HIV test in the last 12 months, 746 (37.0%) tested only once, and 533 (26.4%) took two or more tests. The marginal percentages of individuals taking at least one HIV test was 69.7, 76.7, 53.8, 34.0 and 82.7% for MSM, FSW, PWUD, MCSW, and SNPs, respectively. The marginal percentages of frequent testing was 37.2, 11.7, 23.4, 14.7 and 44.6% for MSM, FSW, PWUD, MCSW, and SNPs, respectively. (Fig. 2).

Socio-demographic characteristics of our study population are as follows. In total, 847 (41.9%) were aged from 18 to 30 years, 1891 (93.5%) were of Han ethnicity, 1114 (55.1%) were from a local household, 1453 (71.9%) had more than 2 years of local living time, 789 (39.0%) had at least a college education level, 1058 (52.3%) had a monthly income less than 3000 CNY, about a half (53.1%) were married/cohabitating, and 1270 (62.8%) indicated never or occasionally drinking alcohol in the past 3 months. (Table 1).

**Table 1:** Socio-demographic and behavioral characteristics of five key populations

Variables	Total N = 2022 (%)	MSM n = 419 (%)	FSW n = 400 (%)	PWUD n = 401 (%)	MCSW n = 400 (%)	SNP n = 402 (%)	P-value
Age (years)							< 0.001
18–30	847 (41.9)	314 (74.9)	105 (26.2)	158 (39.4)	229 (57.2)	41 (10.2)	
31–40	561 (27.7)	54 (12.9)	140 (35.0)	96 (23.9)	130 (32.5)	141 (35.1)	
> 40	610 (30.2)	51 (12.2)	155 (38.8)	145 (36.2)	41 (10.3)	218 (54.2)	
Missing value	4 (0.2)	0 (0.0)	0 (0.0)	2 (0.5)	0 (0.0)	2 (0.5)	
Ethnicity							< 0.001
Han	1891 (93.5)	407 (97.1)	397 (99.3)	395 (98.5)	390 (97.5)	302 (75.1)	
Minorities	126 (6.2)	12 (2.9)	2 (0.5)	6 (1.5)	9 (2.2)	97 (24.1)	
Missing value	5 (0.3)	0 (0.0)	1 (0.2)	0 (0.0)	1 (0.3)	3 (0.8)	
Local household							< 0.001
Yes	1114 (55.1)	148 (35.3)	168 (42.0)	296 (73.8)	215 (53.7)	287 (71.4)	
No	894 (44.2)	271 (64.7)	232 (58.0)	105 (26.2)	182 (45.5)	104 (25.9)	
Missing value	14 (0.7)	0 (0.0)	0 (0.0)	0 (0.0)	3 (0.8)	11 (2.7)	
Local living time							< 0.001
≤ 2 years	563 (27.8)	124 (29.6)	267 (66.7)	49 (12.2)	92 (23.0)	31 (7.7)	
> 2 years	1453 (71.9)	295 (70.4)	133 (33.3)	352 (87.8)	307 (76.7)	366 (91.0)	
Missing value	6 (0.3)	0 (0.0)	0 (0.0)	0 (0.0)	1 (0.3)	5 (1.3)	
Education level							< 0.001
Senior high school and lower	1217 (60.2)	143 (34.1)	372 (93.0)	229 (57.1)	163 (40.7)	310 (77.1)	
College and above	789 (39.0)	276 (65.9)	28 (7.0)	157 (39.2)	237 (59.3)	91 (22.6)	
Missing value	16 (0.8)	0 (0.0)	0 (0.0)	15 (3.7)	0 (0.0)	1 (0.3)	
Monthly income (CNY)							< 0.001
≤ 3000	1058 (52.3)	172 (41.0)	277 (69.2)	209 (52.1)	106 (26.5)	294 (73.1)	
3001–5000	522 (25.8)	123 (29.4)	76 (19.0)	114 (28.4)	124 (31.0)	85 (21.2)	
> 5000	436 (21.6)	124 (29.6)	47 (11.8)	72 (18.0)	170 (42.5)	23 (5.7)	
Missing value	6 (0.3)	0 (0.0)	0 (0.0)	6 (1.5)	0 (0.0)	0 (0.0)	
Marital status							< 0.001
Unmarried/divorced/widowed	940 (46.5)	338 (80.7)	139 (34.7)	279 (69.6)	184 (46.0)	0 (0.0)	
Married/cohabitating	1073 (53.1)	81 (19.3)	261 (65.3)	114 (28.4)	215 (53.7)	402 (100.0)	
Missing value	9 (0.4)	0 (0.0)	0 (0.0)	8 (2.0)	1 (0.3)	0 (0.0)	
AIDS knowledge score^a^							< 0.001
< 6	389 (19.2)	73 (17.4)	65 (16.2)	161 (40.1)	65 (16.2)	25 (6.2)	
≥ 6	1627 (80.5)	346 (82.6)	335 (83.8)	236 (58.9)	334 (83.5)	376 (93.5)	
Missing value	6 (0.3)	0 (0.0)	0 (0.0)	4 (1.0)	1 (0.3)	1 (0.3)	
Alcohol consumption in the past 3 months							< 0.001
Never or occasionally	1270 (62.8)	274 (65.4)	287 (71.8)	230 (57.4)	143 (35.8)	336 (83.6)	
1–4 times a month	406 (20.1)	91 (21.7)	58 (14.5)	73 (18.2)	143 (35.8)	41 (10.2)	
≥ Once a week	342 (16.9)	54 (12.9)	55 (13.7)	95 (23.7)	114 (28.4)	24 (6.0)	
Missing value	4 (0.2)	0 (0.0)	0 (0.0)	3 (0.7)	0 (0.0)	1 (0.2)	

*MSM* men who have sex with men, *FSW* female sex worker, *PWUD* people who use drugs, *MCSW* men who have casual sex with women, *SNP* sero-negative partners among sero-discordant couples

*CNY* Chinese Yuan (1 CNY = 0.1412 USD)

^a^The variable “AIDS knowledge score” was calculated based on responses to eight yes-or-no-or-unclear statements regarding HIV infection and prevention adopted from China national sentinel surveillance questionnaires, which were adapted and tailored for different populations. Each correct answer scored one point with a maximum of eight points. The total points were scaled into two units of analysis: less than six correct answers and six or more correct answers

In terms of high-risk sexual behaviors, 17.7% of MSM had condomless anal sex in the past 6 months, 22.8% of FSWs had condomless commercial sex with male clients in the past month, 35.4% of PWUD had condomless sex after using drugs in the past 12 months, 46.0% of MCSW had condomless sex with FSWs or non-commercial temporary partners in the past 12 months, and 6.0% of SNPs had condomless sex with their sero-positive spouses in the past 12 months. (Table 2).

**Table 2:** Specific high-risk behavioral characteristics for each of five key populations

Variables	MSM n = 419 (%)	FSW n = 400 (%)	PWUD n = 401 (%)	MCSW n = 400 (%)	SNP n = 402 (%)
Condomless anal sex with men in the past 6 months					
No	345 (82.3)	NA	NA	NA	NA
Yes	74 (17.7)	NA	NA	NA	NA
Missing value	0 (0.0)				
Number of male sexual partners in the past 6 months					
< 2	248 (59.2)	NA	NA	NA	NA
≥ 2	171 (40.8)	NA	NA	NA	NA
Missing value	0 (0.0)				
Condomless sex with male clients in the past month					
No	NA	308 (77.0)	NA	NA	NA
Yes	NA	91 (22.8)	NA	NA	NA
Missing value		1 (0.2)			
Number of male clients during a week					
< 7	NA	258 (64.5)	NA	NA	NA
≥ 7	NA	142 (35.5)	NA	NA	NA
Missing value		0 (0.0)			
Ever drug injection					
No	NA	NA	337 (84.0)	NA	NA
Yes	NA	NA	64 (16.0)	NA	NA
Missing value			0 (0.0)		
Frequency of drug use in the past 3 months					
Never or occasionally	NA	NA	272 (67.8)	NA	NA
1–4 times a month	NA	NA	83 (20.7)	NA	NA
≥ once a week	NA	NA	36 (9.0)	NA	NA
Missing value			10 (2.5)		
Condomless sex after using drugs in the past 12 months					
No	NA	NA	257 (64.1)	NA	NA
Yes	NA	NA	142 (35.4)	NA	NA
Missing value			2 (0.5)		
Condomless sex with multiple partners after using drugs in the past 12 months					
No	NA	NA	343 (85.5)	NA	NA
Yes	NA	NA	55 (13.7)	NA	NA
Missing value			3 (0.8)		
Condomless sex with female sex workers/ non-commercial temporary partners in the past 12 months					
No	NA	NA	NA	214 (53.5)	NA
Yes	NA	NA	NA	184 (46.0)	NA
Missing value				2 (0.5)	
Number of female sex workers/non-commercial temporary partners in the past 12 months					
< 2	NA	NA	NA	137 (34.2)	NA
≥ 2	NA	NA	NA	258 (64.5)	NA
Missing value				5 (1.3)	
Condomless sex with HIV-positive spouses in the past 12 months					
No	NA	NA	NA	NA	376 (93.5)
Yes	NA	NA	NA	NA	24 (6.0)
Missing value					2 (0.5)
Frequency of sexual behavior with spouses in the past 12 months					
Never	NA	NA	NA	NA	195 (48.5)
< once a week	NA	NA	NA	NA	113 (28.1)
≥ once a week	NA	NA	NA	NA	94 (23.4)
Missing value					0 (0.0)

*MSM* men who have sex with men, *FSW* female sex worker, *PWUD* people who use drugs, *MCSW* men who have casual sex with women, *SNP* sero-negative partners among sero-discordant couples, *NA* not applicable

### Multivariate PPOM to compare HIV testing frequency among the five key populations

For all five key populations, compared with MSM, FSWs (AOR = 1.97, 95% CI: 1.36–2.86) and SNPs (AOR = 3.63, 95% CI: 2.40–5.49) were more likely to have taken an HIV test, but MCSW (AOR = 0.23, 95% CI: 0.17–0.32) were less likely. Moreover, SNPs (AOR = 4.02, 95% CI: 2.78–5.83) were more likely to be frequent testers, whereas FSWs (AOR = 0.49, 95% CI: 0.32–0.76) and MCSW (AOR = 0.29, 95% CI: 0.20–0.41) were less likely to be frequent testers. Those with higher education (AOR = 1.74, 95% CI: 1.36–2.24), higher AIDS knowledge score (AOR = 1.63, 95% CI: 1.28–2.06), and those who were not from a local household (AOR = 1.44, 95% CI: 1.18–1.75) were more likely to take HIV tests, while those of Non-Han ethnicity (AOR = 0.55, 95% CI: 0.34–0.88) were less likely to take an HIV test. More details are given in Table 3.

**Table 3:** Results of univariate and multivariate PPOMs of five key populations

	Ever testing vs. (No testing)				Frequent testing vs. (Infrequent testing) ^a^			
Variables	OR (95% CI)	P-value	AOR (95% CI)	P-value	OR (95% CI)	P-value	AOR (95% CI)	P-value
Key populations								
MSM	Ref		Ref		Ref		Ref	
FSW	1.44 (1.05,1.96)	0.023	1.97 (1.36,2.86)	< 0.001	0.22 (0.16,0.32)	< 0.001	0.49 (0.32,0.76)	0.001
PWUD	0.51 (0.38,0.67)	< 0.001	0.79 (0.57,1.09)	0.157	0.51 (0.38,0.70)	< 0.001	0.83 (0.59,1.15)	0.265
MCSW	0.22 (0.17,0.30)	< 0.001	0.23 (0.17,0.32)	< 0.001	0.29 (0.21,0.41)	< 0.001	0.29 (0.20,0.41)	< 0.001
SNP	2.08 (1.49,2.90)	< 0.001	3.63 (2.40,5.49)	< 0.001	1.36 (1.03,1.80)	0.032	4.02 (2.78,5.83)	< 0.001
Age (years)								
18–30	Ref		Ref		Ref		Ref	
31–40	0.99 (0.80,1.24)	0.959	0.85 (0.65,1.11)	0.222	0.61 (0.48,0.79)	< 0.001	0.56 (0.41,0.75)	< 0.001
> 40	1.23 (0.99,1.53)	0.067	0.90 (0.68,1.21)	0.496	0.64 (0.51,0.82)	< 0.001	0.58 (0.42,0.80)	< 0.001
Ethnicity								
Han	Ref		Ref		Ref		Ref	
Other	1.29 (0.88,1.91)	0.197	0.55 (0.34,0.88)	0.013	0.65 (0.41,1.02)	0.059	0.31 (0.19,0.50)	< 0.001
Local household								
Yes	Ref		Ref		Ref		Ref	
No	1.38 (1.17,1.62)	< 0.001	1.44 (1.18,1.75)	< 0.001	1.38 (1.17,1.62)	< 0.001	1.44 (1.18,1.75)	< 0.001
Local living time								
≤ 2 years	Ref		Ref		Ref		Ref	
> 2 years	0.71 (0.58,0.88)	0.001	0.91 (0.70,1.19)	0.494	1.75 (1.38,2.22)	< 0.001	1.24 (0.93,1.67)	0.144
Education level								
Senior high school and lower	Ref		Ref		Ref		Ref	
College and above	0.97 (0.81,1.17)	0.761	1.74 (1.36,2.24)	< 0.001	1.98 (1.62,2.42)	< 0.001	1.62 (1.24,2.10)	< 0.001
Monthly income (CNY)								
≤ 3000	Ref		Ref		Ref		Ref	
3001–5000	0.78 (0.62,0.97)	0.025	1.04 (0.81,1.34)	0.773	1.47 (1.16,1.86)	0.001	1.38 (1.06,1.80)	0.016
> 5000	0.53 (0.42,0.67)	< 0.001	0.91 (0.69,1.21)	0.518	1.26 (0.98,1.63)	0.069	1.50 (1.11,2.03)	0.009
Marital status								
Unmarried/divorced/ widowed	Ref				Ref			
Married/cohabitating	1.05 (0.90,1.24)	0.526			1.05 (0.90,1.24)	0.526		
AIDS knowledge score ^b^								
< 6	Ref		Ref		Ref		Ref	
≥ 6	1.94 (1.57,2.39)	< 0.001	1.63 (1.28,2.06)	< 0.001	1.94 (1.57,2.39)	< 0.001	1.63 (1.28,2.06)	< 0.001
Alcohol consumption in the past 3 months								
Never or occasionally	Ref		Ref		Ref		Ref	
1–4 times a month	0.75 (0.61,0.92)	0.005	1.11 (0.88,1.40)	0.381	0.75 (0.61,0.92)	0.005	1.11 (0.88,1.40)	0.381
≥ once a week	0.68 (0.54,0.85)	0.001	1.24 (0.96,1.60)	0.094	0.68 (0.54,0.85)	0.001	1.24 (0.96,1.60)	0.094

*MSM* men who have sex with men, *FSW* female sex worker, *PWUD* people who use drugs, *MCSW* men who have casual sex with women, *SNP* sero-negative partners among sero-discordant couples, *OR* odds ratio, *CI* confidence interval, *CNY* Chinese Yuan (1 CNY = 0.1412 USD)

*NA* Not applicable, which indicated the P-value of the particular variable in univariate analysis was ≥ 0.05

^a^Consistent parameters in the “Frequent testing vs. Infrequent testing” and “Ever testing vs. No testing” indicated that the effect of the particular variable was symmetrical across categories of HIV testing frequency, which could be regarded that proportional odds assumption was not violated

^b^The variable “AIDS knowledge score” was calculated based on responses to eight yes-or-no-or-unclear statements regarding HIV infection and prevention adopted from China national sentinel surveillance questionnaires, which were adapted and tailored for different populations. Each correct answer scored one point with a maximum of eight points. The total points were scaled into two units of analysis: less than six correct answers and six or more correct answers

### Factors associated with HIV testing frequency for each key population

Results of the univariate and multivariate PPOMs for each key population is displayed in Table S7-S11 of Additional file 3.

For MSM, those with higher education level (AOR = 1.61, 95% CI: 1.08–2.41), higher AIDS knowledge score (AOR = 2.08, 95% CI: 1.27–3.40), and those reporting having condomless anal sex with men in the past 6 months (AOR = 2.42, 95% CI: 1.51–3.87) were more likely to test for HIV and to be frequent testers at that (Additional file 3: Table S7).

For FSWs, those aged 31–40 (AOR = 2.50, 95% CI: 1.30–4.81) or > 40 (AOR = 2.24, 95% CI: 1.16–4.34), who were not from a local household (AOR = 2.01, 95% CI: 1.13–3.59), who had a higher AIDS knowledge score (AOR = 2.48, 95% CI: 1.28–4.81) and reported drinking alcohol more than once a week (AOR = 2.87, 95% CI: 1.19–6.94) were more likely to take an HIV test. But those with higher education level (AOR = 0.23, 95% CI: 0.10–0.55) were less likely to take HIV testing. Compared with participants whose monthly income > 5000 CNY, those with ≤ 3000 CNY were more likely to have taken an HIV test, but less likely to be a frequent HIV tester (AOR = 0.38, 95% CI: 0.17–0.83; AOR = 2.66, 95% CI: 1.03–6.87). Those who reported ≥ 7 male clients during a week (AOR = 3.11, 95% CI: 1.46–6.60) were more likely to be frequent testers (Additional file 3: Table S8).

For PWUD, those aged 31–40 (AOR = 0.38, 95% CI: 0.19–0.75) or > 40 (AOR = 0.39, 95% CI: 0.19–0.80) and married/cohabitating participants (AOR = 0.28, 95% CI: 0.16–0.48) were less likely to have taken an HIV test and also less likely to be a frequent tester. On the other hand, those with higher AIDS knowledge score (AOR = 1.77, 95% CI: 1.10–2.86) and who had condomless sex with multiple partners after using drugs in the past year (AOR = 2.37, 95% CI: 1.18–4.75) were more likely to have taken an HIV test and to be a frequent tester. Compared with PWUD whose monthly income is ≤ 3000 CNY, those at an income of 3001–5000 CNY (AOR = 2.04, 95% CI: 1.09–3.82) were more like to have taken an HIV test, and those with > 5000 CNY (AOR = 2.18, 95% CI: 1.02–4.68) were more likely to be frequent HIV testers. Those who reported alcohol use at a rate of more than once a week (AOR = 2.17, 95% CI: 1.15–4.12) were more likely to have taken an HIV test (Additional file 3: Table S9).

For MCSW, those who reported drinking alcohol at a rate of 1–4 times a month (AOR = 0.53, 95% CI: 0.32–0.88) were less likely to have taken one or more HIV tests in the past year, relative to non-drinkers (Additional file 3: Table S10).

For SNPs, individuals of non-Han ethnicity (AOR = 0.42, 95% CI: 0.23–0.77) and those who were not from a local household (AOR = 0.55, 95% CI: 0.30–0.99) were less likely to have taken an HIV test. Those who were frequent HIV testers were more likely to have a higher education level (AOR = 2.67, 95% CI: 1.45–4.90) but less likely to be of a non-Han ethnicity (AOR = 0.18, 95% CI: 0.09–0.35) or to have reported engaging in sexual activity with their sero-positive spouses at a rate of less than once a week (AOR = 0.56, 95% CI: 0.33–0.96) or more than once a week (AOR = 0.39, 95% CI: 0.21–0.72) in the past year (Additional file 3: Table S11).

## Discussion

The purposes of this study was to explore HIV testing frequencies amongst five key populations in ten cities of China. Specifically, we explored the socio-demographic and behavioral factors associated with HIV testing frequencies using PPOMs from two perspectives, one considering whether individuals had taken an HIV test in the past 12 months, and one considering whether individuals had taken two or more HIV tests, which we refer to as being a frequent HIV tester.

The percentages of individuals reporting to have taken an HIV testing in the past 12 months differed amongst the five key populations. As compared with MSM, FSWs and SNPs were more likely to have taken an HIV test, while MCSW were less likely. Interestingly, again relative to MSM, we found that FSWs were less likely to be frequent HIV testers even though they were more likely to have taken an HIV test. The HIV testing uptake rate for MSM in the past 12 months was 69.7% in our study, which is significantly higher than the 38% found in a 2012 meta-analysis [25]. This may be attributable to the Chinese government strengthening its public health efforts in promoting HIV testing in recent years. SNPs were the most proactive group of HIV testers, which is possibly because sero-positive people are obligated to disclose to their spouses according to Chinese Regulation on the Prevention and Treatment of HIV/ AIDS. A study in Yunnan province of China found 76.32% of SNPs took an HIV test within 3 months after their spouse confirmed an HIV infection [26]. For MCSW, the proportion of HIV testing was lower than others at 34%. Similarly, a study in 2019 reflected the low uptake of HIV testing (23.8%) among male clients of FSWs in China [16]. This may be due to commercial sex being illegal in China [27] and the hard-to-locate nature of this population. Future interventions and HIV testing promotion efforts should focus on this population. For FSWs, 76.8% reported testing for HIV in the past 12 months in our study, which was higher than the 48% found in a study by Chow in 2015 [28]. At the same time, only 11.7% of FSWs in our study could be characterized as frequent testers. Complex psychosocial and demographic characteristics may affect their testing behavior, some of which are interpreted in detail below.

Stratified analyses showed that certain socio-demographic factors inconsistently influenced HIV testing frequency among the different populations. For MSM and SNPs, those with higher education were more likely to have taken an HIV test, which is consistent with previous studies [29, 30]. People with higher education are more likely to have access to resources and thus more likely to see the benefits of knowing their HIV status. Conversely, we found that FSWs with higher education were less likely to have taken an HIV test, which is consistent with a study in Southwest China [31]. A possible explanation was that “active testing” conducted by local CDC offices had targeted certain commercial sex venues, such as those that provide more “explicit” commercial sex services (e.g., massage parlors and streets). In other venues such as hotels and night clubs, where commercial sex is hidden under the cover of other services, the outreach for “active testing” is less common. And the literature suggests that the FSW with less education are more likely to work in venues that offer more “explicit” commercial sex service [32].

The impact of monthly income on HIV testing frequency varied for FSWs and PWUD. We found FSWs who earned ≤ 3000 CNY monthly to be more likely to take at least one HIV test but to be less likely to be frequent HIV testers compared with their counterparts earning > 5000 CNY. Low-income FSWs are more likely to report high rate of condomless sex with their clients (on occasion, sacrificing condom use for extra payment) and are known to suffer severe stigmatization and marginalization in society [33]. More frequent high-risk sexual experiences may compel them to test for HIV but limited resources, due to poverty, may prevent them to being frequent HIV testers [34]. PWUD with higher income were more likely to take at least one HIV test and to be considered frequent testers. It is possible that more disposable income enables them to more easily access illicit drugs and report high-risk sexual behavior after using them [35].

With respect to age, we found that elder PWUD were less likely to have reported an HIV test in the past 12 months, which is consistent with a study in Shanghai [36]. Younger people tend to engage more frequently in high-risk activities but also have a greater chance of accessing HIV-related information. However, our study found HIV testing rates to be higher amongst elder FSWs. Working in the sex industry for a longer period and starting sex work at older ages were reported to be associated with higher HIV testing uptake [34], which suggests that the willingness to take an HIV test may increase with time and correlate closely with HIV risk perception among FSWs.

Evidence shows that heavy alcohol use can impair judgment and directly affect the brain resulting in reduced inhibitions and diminished risk perception [37]. In addition, alcohol consumption in entertainment venues such as karaoke bars, dancing hall, nightclubs, is sometimes accompanied by illicit drug use and unwanted sexual behaviors [38], which increases the likelihood of HIV transmission. In our study, associations with alcohol use were mixed. We found more frequent alcohol consumption appeared to facilitate HIV testing among FSWs and PWUD, but actually decreased the rate of HIV testing among MCSW.

In this study, high-risk sexual behaviors among certain key populations were found to be linked to more frequent HIV testing, which is consistent with studies in London and Germany [9, 11]. Specifically, we found that MSM who had condomless anal sex with men in the past 6 months, FSWs who had ≥ 7 male clients during a week, and PWUD who had condomless sex with multiple partners after using drugs in the past year were all more likely to be frequent HIV testers. Of course, from a public health perspective, the desired goal is to reduce high-risk behaviors, not strictly to increase the frequency of HIV testing. So individuals pursuing high-risk behaviors would surely benefit from other interventions such as public relations campaigns on the importance of condom use preventing HIV transmission as well as the potential benefits of biochemical approaches such as non-occupational post-exposure prophylaxis (nPEP) and pre-exposure prophylaxis (PrEP) [39, 40].

Several limitations of this study should be acknowledged. First, self-reported data, some of which is sensitive (e.g., sexual behavior, illicit drug use), may not always be accurately reported. Second, a convenience sampling approach was used to recruit participants, which may impact the HIV testing percentages that would have been reported under a (much less feasible) probability-based sampling approach. Third, we only focused on comparing the socio-demographic and behavioral factors associated with HIV testing frequency in this study without taking structural factors (e.g., stigma) into consideration. Fourth, we are unable to distinguish between HIV self-testing and facility-based testing in this study. It is possible that results and insights could differ if we were instead able to make that distinction for our recruited participants [41].

## Conclusions

Rates of HIV testing varied amongst the five key populations considered in our study. Compared with MSM, MCSW were less likely to have reported taking an HIV test within the last 12 months, while SNPs were more likely to be frequent HIV testers and FSWs were more likely to take have taken an HIV test but less likely to be frequent HIV testers. The factors associated with HIV testing frequency were not completely consistent for each population. In order to optimize HIV prevention and care, it is important to identify high-risk subgroups reporting low rates of HIV testing. That said, public health practitioners should also focus on behavioral change for individuals in high-risk subgroups even if data demonstrates they tend to more frequently test for HIV. Intensive and tailored behavioral interventions coupled with biochemical prophylaxis would be helpful in that regard.

## Supplementary Information

**Additional file 1.** Sample size calculation.

**Additional file 2.** Score test for the proportional odds assumption of all the variables in PPOMs.

**Additional file 3.** Results of univariate and multivariate analysis using HIV testing frequency as response with three ordered categories for each of key population.

## Data Availability

The datasets used and/or analysed during the current study are available from the corresponding author (Wei Ma, weima@sdu.edu.cn) on reasonable request.

## Abbreviations

AOR: Adjusted odds ratio
CBO: Community-based organization
CDC: Centre for Disease Control and Prevention
CI: Confidence interval
CNY: Chinese Yuan
FSW: Female sex worker
MCSF: Men who have casual sex with female
MSM: Men who have sex with men
nPEP: Non-occupational post-exposure prophylaxis
PPOM: Partial proportional odds model
PrEP: Pre-exposure prophylaxis
PWUD: People who use drugs
SNP: Sero-negative partner among sero-discordant couples
USD: United States dollar
