# Supplementary material for: HIV testing frequency and associated factors among five key populations in ten cities of China: a cross-sectional study
Source: BMC Infect Dis. 2022 Feb 28;22:195. doi: 10.1186/s12879-022-07189-6 (PMC8883696; doi:10.1186/s12879-022-07189-6)
Supplement: Supplementary file 1 — Additional file 1. Sample size calculation. [file 12879_2022_7189_MOESM1_ESM.docx]

**Additional file 1. Sample size calculation**

We targeted a sample size of 400 participants for each high-risk population, which was calculated based on perceptions of non-occupation post-exposure prophylaxis (nPEP) because this study was sourced from a feasibility study of nPEP in China. To verify this was sufficient to achieve our desired precision goals of HIV testing, we recalculated the sample size according to the following formula:

$$n=\frac{Z_{\alpha}^{2}}{d^{2}}p\left( 1-p \right)$$

We presumed the rates of HIV testing uptake of five key populations through literature review (1-5), namely p=0.56 for MSM, 0.48 for FSWs, 0.26 for DUs, 0.24 for MCSW, 0.76 for SNPs, and simultaneously we assumed Zα=1.96, d=0.2p, the required sample size was 76 for MSM, 105 for FSWs, 274 for DUs, 305 for MCSW, and 31 for SNPs. The existing sample size met the requirement of the current study.

**References**

1. Li R, Pan X, Ma Q, Wang H, He L, Jiang T, et al. Prevalence of prior HIV testing and associated factors among MSM in Zhejiang Province, China: a cross-sectional study. BMC Public Health. 2016; 16(1): 1152.

2. Hong Y, Zhang C, Li X, Fang X, Lin X, Zhou Y, et al. HIV testing behaviors among female sex workers in Southwest China. AIDS Behav. 2012; 16(1): 44-52.

3. Jiang Z, Xiu C, Yang J, Zhang X, Liu M, Chen X, et al. HIV test uptake and related factors amongst heterosexual drug users in Shandong province, China. PLoS One. 2018; 13(10): e204489.

4. Fang Y, Zhang Y, Wang Z, Ip M, Li J, Lau JTF. Low uptake of HIV testing among male clients of female sex workers in China. AIDS Care. 2019; 31(2): 193-198.

5. Yu H, Han Y, Shi Y, Huo J, Zhang X, Yang Y, et al. Case-control study of the correlation of partner notification and HIV testing with seroconversion of spouses among human immunodeficiency virus sero-discordant couples. Chinese Journal of AIDS & STD. 2017; 23(10): 898-900. (In Chinese)
