# Supplementary material for: HIV testing frequency and associated factors among five key populations in ten cities of China: a cross-sectional study
Source: BMC Infect Dis. 2022 Feb 28;22:195. doi: 10.1186/s12879-022-07189-6 (PMC8883696; doi:10.1186/s12879-022-07189-6)
Supplement: Supplementary file 2 — Additional file 2. Score test for the proportional odds assumption of all the variables in PPOMs. [file 12879_2022_7189_MOESM2_ESM.docx]

**Additional file 2. Score test for the proportional odds assumption of all the variables in PPOMs**

**Table S1. Score test for the proportional odds assumption of all the variables in PPOM among five key populations**

| **Variables** | **Chi-square** | ***P*-value** |
| --- | --- | --- |
| Key population | 129.717 | < 0.001 |
| Age (years) | 33.145 | < 0.001 |
| Ethnicity | 7.718 | 0.005 |
| Local household | 2.815 | 0.093 |
| Local living time | 50.856 | < 0.001 |
| Education level | 51.317 | < 0.001 |
| Monthly income | 60.318 | < 0.001 |
| Marital status | 1.752 | 0.186 |
| AIDS knowledge score | 2.630 | 0.104 |
| Alcohol consumption in the past 3 months | 5.871 | 0.053 |
| *Note*: A significant test (p < 0.05) indicates that the proportional odds assumption has been violated. | | |

**Table S2. Score test for the proportional odds assumption of all the variables in PPOM among MSM**

| **Variables** | **Chi-square** | ***P*-value** |
| --- | --- | --- |
| Age (years) | 6.445 | 0.039 |
| Ethnicity | 2.247 | 0.134 |
| Local household | 0.523 | 0.469 |
| Local living time | 0.351 | 0.553 |
| Education level | 1.236 | 0.266 |
| Monthly income | 1.343 | 0.511 |
| Marital status | 0.019 | 0.891 |
| AIDS knowledge score | < 0.001 | 0.999 |
| Alcohol consumption in the past 3 months | 0.597 | 0.742 |
| Condomless anal sex with men in the past 6 months | 2.557 | 0.110 |
| Number of male sexual partners in the past 6 months | 0.815 | 0.367 |
| *Note*: A significant test (p < 0.05) indicates that the proportional odds assumption has been violated. | | |

**Table S3. Score test for the proportional odds assumption of all the variables in PPOM among FSWs**

| **Variables** | **Chi-square** | ***P*-value** |
| --- | --- | --- |
| Age (years) | 18.252 | < 0.001 |
| Ethnicity | 0.080 | 0.778 |
| Local household | 8.100 | 0.004 |
| Local living time | 18.441 | < 0.001 |
| Education level | 3.119 | 0.077 |
| Monthly income | 40.042 | < 0.001 |
| Marital status | 2.201 | 0.138 |
| AIDS knowledge score | 20.753 | < 0.001 |
| Alcohol consumption in the past 3 months | 18.482 | < 0.001 |
| Condomless sex with male clients in the past month | 3.381 | 0.066 |
| Number of male clients during a week | 22.322 | < 0.001 |
| *Note*: A significant test (p < 0.05) indicates that the proportional odds assumption has been violated. | | |

**Table S4. Score test for the proportional odds assumption of all the variables in PPOM among PWUD**

| **Variables** | **Chi-square** | ***P*-value** |
| --- | --- | --- |
| Age (years) | 2.713 | 0.258 |
| Ethnicity | 0.255 | 0.613 |
| Local household | 1.729 | 0.189 |
| Local living time | 0.101 | 0.751 |
| Education level | 0.389 | 0.533 |
| Monthly income | 21.227 | < 0.001 |
| Marital status | 3.001 | 0.083 |
| AIDS knowledge score | 1.723 | 0.189 |
| Alcohol consumption in the past 3 months | 21.051 | < 0.001 |
| Ever drug injection | 10.543 | 0.001 |
| Frequency of drug use in the past 3 months | 0.501 | 0.779 |
| Condomless sex after using drugs in the past 12 months | 0.957 | 0.328 |
| Condomless sex with multiple partners after using drugs in the past 12 months | 0.382 | 0.537 |
| *Note*: A significant test (p < 0.05) indicates that the proportional odds assumption has been violated. | | |

**Table S5. Score test for the proportional odds assumption of all the variables in PPOM among MCSW**

| **Variables** | **Chi-square** | ***P*-value** |
| --- | --- | --- |
| Age (years) | 3.329 | 0.189 |
| Ethnicity | 0.845 | 0.358 |
| Local household | 0.006 | 0.939 |
| Local living time | 0.935 | 0.334 |
| Education level | 0.611 | 0.435 |
| Monthly income | 2.276 | 0.320 |
| Marital status | 1.866 | 0.172 |
| AIDS knowledge score | 0.597 | 0.440 |
| Alcohol consumption in the past 3 months | 0.851 | 0.654 |
| Condomless sex with female sex worker/ non-commercial temporary partner in the past 12 months | 0.023 | 0.878 |
| Number of female sex workers/non-commercial temporary partners in the past 12 months | 0.394 | 0.530 |
| *Note*: A significant test (p < 0.05) indicates that the proportional odds assumption has been violated. | | |

**Table S6. Score test for the proportional odds assumption of all the variables in PPOM among SNPs**

| **Variables** | **Chi-square** | ***P*-value** |
| --- | --- | --- |
| Age (years) | 2.763 | 0.251 |
| Ethnicity | 10.883 | 0.001 |
| Local household | 11.386 | 0.001 |
| Local living time | 0.038 | 0.846 |
| Education level | 4.363 | 0.037 |
| Monthly income | 3.575 | 0.167 |
| Marital status | 0.718 | 0.397 |
| AIDS knowledge score | 7.320 | 0.007 |
| Alcohol consumption in the past 3 months | 1.807 | 0.405 |
| Condomless sex with spouse in the past 12 months | 0.009 | 0.924 |
| Frequencies of sexual behavior with spouse in the past 12 months | 32.280 | < 0.001 |
| *Note*: A significant test (p < 0.05) indicates that the proportional odds assumption has been violated. | | |
