# Supplementary material for: HIV testing frequency and associated factors among five key populations in ten cities of China: a cross-sectional study
Source: BMC Infect Dis. 2022 Feb 28;22:195. doi: 10.1186/s12879-022-07189-6 (PMC8883696; doi:10.1186/s12879-022-07189-6)
Supplement: Supplementary file 3 — Additional file 3. Results of univariate and multivariate analysis using HIV testing frequency as response with three ordered categories for each of key population. [file 12879_2022_7189_MOESM3_ESM.docx]

**Additional file 3. Results of univariate and multivariate analysis using HIV testing frequency as response with three ordered categories for each of key population**

**Table S7. Results of univariate and multivariate PPOMs of MSM**

|  | **Ever testing vs. (No testing)** | | | | **Frequent testing vs. (Infrequent testing) ^a^** | | | |
| --- | --- | --- | --- | --- | --- | --- | --- | --- |
| **Variables** | **OR (95%CI)** | **P-value** | **AOR (95%CI)** | **P-value** | **OR (95%CI)** | **P-value** | **AOR (95%CI)** | **P-value** |
| **Age (years)** |  |  |  |  |  |  |  |  |
| 18-30 | Ref |  | Ref |  | Ref |  | Ref |  |
| 31-40 | 0.96 (0.51,1.79) | 0.894 | 0.72 (0.37,1.37) | 0.313 | 0.53 (0.28,1.01) | 0.055 | 0.42 (0.22,0.82) | 0.011 |
| >40 | 1.16 (0.60,2.25) | 0.652 | 1.07 (0.53,2.17) | 0.853 | 0.76 (0.41,1.41) | 0.380 | 0.69 (0.35,1.34) | 0.268 |
| **Ethnicity** |  |  |  |  |  |  |  |  |
| Han | Ref |  |  |  | Ref |  |  |  |
| Other | 0.85 (0.30,2.45) | 0.768 |  |  | 0.85 (0.30,2.45) | 0.768 |  |  |
| **Local household** |  |  |  |  |  |  |  |  |
| Yes | Ref |  |  |  | Ref |  |  |  |
| No | 1.13 (0.78,1.63) | 0.524 |  |  | 1.13 (0.78,1.63) | 0.524 |  |  |
| **Local living time** |  |  |  |  |  |  |  |  |
| ≤ 2 years | Ref |  |  |  | Ref |  |  |  |
| > 2 years | 1.25 (0.85,1.83) | 0.266 |  |  | 1.25 (0.85,1.83) | 0.266 |  |  |
| **Education level** |  |  |  |  |  |  |  |  |
| Senior high school and lower | Ref |  | Ref |  | Ref |  | Ref |  |
| College and above | 1.65 (1.14,2.40) | 0.009 | 1.61 (1.08,2.41) | 0.019 | 1.65 (1.14,2.40) | 0.009 | 1.61 (1.08,2.41) | 0.019 |
| **Monthly income (CNY)** |  |  |  |  |  |  |  |  |
| ≤ 3000 | Ref |  |  |  | Ref |  |  |  |
| 3001-5000 | 1.05 (0.69,1.61) | 0.812 |  |  | 1.05 (0.69,1.61) | 0.812 |  |  |
| > 5000 | 1.17 (0.76,1.79) | 0.477 |  |  | 1.17 (0.76,1.79) | 0.477 |  |  |
| **Marital status** |  |  |  |  |  |  |  |  |
| Unmarried/divorced/ widowed | Ref |  |  |  | Ref |  |  |  |
| Married/cohabitating | 0.77 (0.49,1.20) | 0.251 |  |  | 0.77 (0.49,1.20) | 0.251 |  |  |
| **AIDS knowledge score ^b^** |  |  |  |  |  |  |  |  |
| < 6 | Ref |  | Ref |  | Ref |  | Ref |  |
| ≥ 6 | 2.21 (1.38,3.55) | 0.001 | 2.08 (1.27,3.40) | 0.004 | 2.21 (1.38,3.55) | 0.001 | 2.08 (1.27,3.40) | 0.004 |
| **Alcohol consumption in the past 3 months** |  |  |  |  |  |  |  |  |
| Never or occasionally | Ref |  |  |  | Ref |  |  |  |
| 1-4 times a month | 1.28 (0.83,1.98) | 0.271 |  |  | 1.28 (0.83,1.98) | 0.271 |  |  |
| ≥ once a week | 1.26 (0.73,2.15) | 0.407 |  |  | 1.26 (0.73,2.15) | 0.407 |  |  |
| **Condomless anal sex with men in the past 6 months** |  |  |  |  |  |  |  |  |
| No | Ref |  | Ref |  |  |  | Ref |  |
| Yes | 2.86 (1.92,4.24) | <0.001 | 2.42 (1.51,3.87) | <0.001 | 2.86 (1.92,4.24) | <0.001 | 2.42 (1.51,3.87) | <0.001 |
| **Number of male sexual partners in the past 6 months** |  |  |  |  |  |  |  |  |
| < 2 | Ref |  | Ref |  |  |  | Ref |  |
| ≥ 2 | 2.09 (1.45,3.01) | <0.001 | 1.46 (0.94,2.26) | 0.092 | 2.09 (1.45,3.01) | <0.001 | 1.46 (0.94,2.26) | 0.092 |
| *Note*: CI=confidence interval; CNY=Chinese Yuan (1 CNY=0.1412 USD)  NA=Not applicable, which indicated the P-value of the particular variable in univariate analysis was ≥0.05  ^a^ Consistent parameters in the “Frequent testing vs. Infrequent testing” and “Ever testing vs. No testing” indicated that the effect of the particular variable was symmetrical across categories of HIV testing frequency, which could be regarded that proportional odds assumption was not violated.  ^b^ The variable “AIDS knowledge score” was calculated based on responses to eight yes-or-no-or-unclear statements regarding HIV infection and prevention adopted from China national sentinel surveillance questionnaires, which were adapted and tailored for different populations. Each correct answer scored one point with a maximum of eight points. The total points were scaled into two units of analysis: less than six correct answers and six or more correct answers. | | | | | | | | |

**Table S8. Results of univariate and multivariate PPOMs of FSW**

|  | **Ever testing vs. (No testing)** | | | | **Frequent testing vs. (Infrequent testing) ^a^** | | | |
| --- | --- | --- | --- | --- | --- | --- | --- | --- |
| **Variables** | **OR (95%CI)** | **P-value** | **AOR (95%CI)** | **P-value** | **OR (95%CI)** | **P-value** | **AOR (95%CI)** | **P-value** |
| **Age (years)** |  |  |  |  |  |  |  |  |
| 18-30 | Ref |  | Ref |  | Ref |  | Ref |  |
| 31-40 | 2.18 (1.22,3.91) | 0.009 | 2.50 (1.30,4.81) | 0.006 | 0.67 (0.33,1.37) | 0.270 | 0.95 (0.42,2.12) | 0.895 |
| >40 | 2.17 (1.22,3.83) | 0.007 | 2.24 (1.16,4.34) | 0.017 | 0.41 (0.19,0.88) | 0.023 | 0.67 (0.26,1.71) | 0.398 |
| **Ethnicity** |  |  |  |  |  |  |  |  |
| Han | Ref |  |  |  | Ref |  |  |  |
| Other | 8.21 (0.53,128.00) | 0.133 |  |  | 8.21 (0.53,128.00) | 0.133 |  |  |
| **Local household** |  |  |  |  |  |  |  |  |
| Yes | Ref |  | Ref |  | Ref |  | Ref |  |
| No | 1.76 (1.10,2.81) | 0.018 | 2.01 (1.13,3.59) | 0.018 | 0.66 (0.36,1.22) | 0.182 | 0.88 (0.41,1.89) | 0.745 |
| **Local living time** |  |  |  |  |  |  |  |  |
| ≤ 2 years | Ref |  | Ref |  | Ref |  | Ref |  |
| > 2 years | 0.46 (0.28,0.73) | 0.001 | 0.82 (0.46,1.45) | 0.496 | 1.92 (1.04,3.55) | 0.038 | 1.73 (0.87,3.44) | 0.121 |
| **Education level** |  |  |  |  |  |  |  |  |
| Senior high school and lower | Ref |  | Ref |  | Ref |  | Ref |  |
| College and above | 0.18 (0.08,0.40) | <0.001 | 0.23 (0.10,0.55) | 0.001 | 0.18 (0.08,0.40) | <0.001 | 0.23 (0.10,0.55) | 0.001 |
| **Monthly income (CNY)** |  |  |  |  |  |  |  |  |
| ≤ 3000 | Ref |  | Ref |  | Ref |  | Ref |  |
| 3001-5000 | 0.42 (0.24,0.75) | 0.003 | 0.51 (0.26,1.01) | 0.052 | 1.87 (0.87,4.03) | 0.111 | 0.89 (0.37,2.18) | 0.804 |
| > 5000 | 0.39 (0.20,0.76) | 0.006 | 0.38 (0.17,0.83) | 0.016 | 4.22 (1.96,9.11) | <0.001 | 2.66 (1.03,6.87) | 0.043 |
| **Marital status** |  |  |  |  |  |  |  |  |
| Unmarried/divorced/ widowed | Ref |  |  |  | Ref |  |  |  |
| Married/cohabitating | 1.34 (0.88,2.05) | 0.171 |  |  | 1.34 (0.88,2.05) | 0.171 |  |  |
| **AIDS knowledge score ^b^** |  |  |  |  |  |  |  |  |
| < 6 | Ref |  | Ref |  | Ref |  | Ref |  |
| ≥ 6 | 2.07 (1.17,3.68) | 0.013 | 2.48 (1.28,4.81) | 0.007 | 0.40 (0.20,0.80) | 0.009 | 0.87 (0.39,1.96) | 0.737 |
| **Alcohol consumption in the past 3 months** |  |  |  |  |  |  |  |  |
| Never or occasionally | Ref |  | Ref |  | Ref |  | Ref |  |
| 1-4 times a month | 0.84 (0.44,1.61) | 0.597 | 1.12 (0.53,2.38) | 0.766 | 1.93 (0.81,4.57) | 0.137 | 1.03 (0.37,2.85) | 0.957 |
| ≥ once a week | 0.95 (0.48,1.87) | 0.873 | 2.87 (1.19,6.94) | 0.019 | 5.39 (2.63,11.05) | <0.001 | 1.63 (0.61,4.41) | 0.333 |
| **Condomless sex with male clients in the past month** |  |  |  |  |  |  |  |  |
| No | Ref |  |  |  | Ref |  |  |  |
| Yes | 1.16 (0.72,1.87) | 0.552 |  |  | 1.16 (0.72,1.87) | 0.552 |  |  |
| **Number of male clients during a week** |  |  |  |  |  |  |  |  |
| < 7 | Ref |  | Ref |  | Ref |  | Ref |  |
| ≥ 7 | 0.89 (0.55,1.43) | 0.624 | 1.07 (0.61,1.89) | 0.805 | 4.71 (2.45,9.06) | <0.001 | 3.11 (1.46,6.60) | 0.003 |
| *Note*: CI=confidence interval; CNY=Chinese Yuan (1 CNY=0.1412 USD)  NA=Not applicable, which indicated the P-value of the particular variable in univariate analysis was ≥0.05  ^a^ Consistent parameters in the “Frequent testing vs. Infrequent testing” and “Ever testing vs. No testing” indicated that the effect of the particular variable was symmetrical across categories of HIV testing frequency, which could be regarded that proportional odds assumption was not violated.  ^b^ The variable “AIDS knowledge score” was calculated based on responses to eight yes-or-no-or-unclear statements regarding HIV infection and prevention adopted from China national sentinel surveillance questionnaires, which were adapted and tailored for different populations. Each correct answer scored one point with a maximum of eight points. The total points were scaled into two units of analysis: less than six correct answers and six or more correct answers. | | | | | | | | |

**Table S9. Results of univariate and multivariate PPOMs of PWUD**

|  | **Ever testing vs. (No testing)** | | | | **Frequent testing vs. (Infrequent testing) ^a^** | | | |
| --- | --- | --- | --- | --- | --- | --- | --- | --- |
| **Variables** | **OR (95%CI)** | **P-value** | **AOR (95%CI)** | **P-value** | **OR (95%CI)** | **P-value** | **AOR (95%CI)** | **P-value** |
| **Age (years)** |  |  |  |  |  |  |  |  |
| 18-30 | Ref |  | Ref |  | Ref |  | Ref |  |
| 31-40 | 0.18 (0.11,0.29) | <0.001 | 0.38 (0.19,0.75) | 0.005 | 0.18 (0.11,0.29) | <0.001 | 0.38 (0.19,0.75) | 0.005 |
| >40 | 0.13 (0.08,0.21) | <0.001 | 0.39 (0.19,0.80) | 0.010 | 0.13 (0.08,0.21) | <0.001 | 0.39 (0.19,0.80) | 0.010 |
| **Ethnicity** |  |  |  |  |  |  |  |  |
| Han | Ref |  | Ref |  | Ref |  | Ref |  |
| Other | 6.27 (1.20,32.82) | 0.030 | 3.16 (0.53,19.06) | 0.209 | 6.27 (1.20,32.82) | 0.030 | 3.16 (0.53,19.06) | 0.209 |
| **Local household** |  |  |  |  |  |  |  |  |
| Yes | Ref |  | Ref |  | Ref |  | Ref |  |
| No | 3.96 (2.57,6.08) | <0.001 | 1.74 (0.95,3.19) | 0.074 | 3.96 (2.57,6.08) | <0.001 | 1.74 (0.95,3.19) | 0.074 |
| **Local living time** |  |  |  |  |  |  |  |  |
| ≤ 2 years | Ref |  | Ref |  | Ref |  | Ref |  |
| > 2 years | 0.55 (0.32,0.96) | 0.035 | 1.58 (0.77,3.25) | 0.215 | 0.55 (0.32,0.96) | 0.035 | 1.58 (0.77,3.25) | 0.215 |
| **Education level** |  |  |  |  |  |  |  |  |
| Senior high school and lower | Ref |  | Ref |  | Ref |  | Ref |  |
| College and above | 3.51 (2.37,5.21) | <0.001 | 1.48 (0.84,2.60) | 0.179 | 3.51 (2.37,5.21) | <0.001 | 1.48 (0.84,2.60) | 0.179 |
| **Monthly income (CNY)** |  |  |  |  |  |  |  |  |
| ≤ 3000 | Ref |  | Ref |  | Ref |  | Ref |  |
| 3001-5000 | 1.92 (1.20,3.09) | 0.007 | 2.04 (1.09,3.82) | 0.026 | 1.86 (1.08,3.21) | 0.025 | 1.71 (0.87,3.34) | 0.120 |
| > 5000 | 0.90 (0.52,1.53) | 0.685 | 1.05 (0.51,2.17) | 0.897 | 2.39 (1.30,4.39) | 0.005 | 2.18 (1.02,4.68) | 0.045 |
| **Marital status** |  |  |  |  |  |  |  |  |
| Unmarried/divorced/ widowed | Ref |  | Ref |  | Ref |  | Ref |  |
| Married/cohabitating | 0.34 (0.22,0.52) | <0.001 | 0.28 (0.16,0.48) | <0.001 | 0.34 (0.22,0.52) | <0.001 | 0.28 (0.16,0.48) | <0.001 |
| **AIDS knowledge score ^b^** |  |  |  |  |  |  |  |  |
| < 6 | Ref |  | Ref |  | Ref |  | Ref |  |
| ≥ 6 | 1.50 (1.03,2.20) | 0.035 | 1.77 (1.10,2.86) | 0.018 | 1.50 (1.03,2.20) | 0.035 | 1.77 (1.10,2.86) | 0.018 |
| **Alcohol consumption in the past 3 months** |  |  |  |  |  |  |  |  |
| Never or occasionally | Ref |  | Ref |  | Ref |  | Ref |  |
| 1-4 times a month | 2.55 (1.46,4.45) | 0.001 | 1.89 (0.96,3.72) | 0.065 | 1.29 (0.72,2.32) | 0.386 | 1.20 (0.59,2.43) | 0.615 |
| ≥ once a week | 1.92 (1.18,3.13) | 0.009 | 2.17 (1.15,4.12) | 0.018 | 0.52 (0.27,0.99) | 0.045 | 0.65 (0.29,1.42) | 0.277 |
| **Ever drug injection** |  |  |  |  |  |  |  |  |
| No | Ref |  | Ref |  | Ref |  | Ref |  |
| Yes | 0.78 (0.45,1.32) | 0.351 | 1.64 (0.80,3.35) | 0.178 | 0.13 (0.04,0.44) | <0.001 | 0.46 (0.10,2.11) | 0.317 |
| **Frequency of drug use in the past 3 months** |  |  |  |  |  |  |  |  |
| Never or occasionally | Ref |  | Ref |  | Ref |  | Ref |  |
| 1-4 times a month | 3.33 (2.09,5.31) | <0.001 | 1.74 (0.97,3.11) | 0.061 | 3.33 (2.09,5.31) | <0.001 | 1.74 (0.97,3.11) | 0.061 |
| ≥ once a week | 2.01 (1.06,3.83) | 0.034 | 0.79 (0.37,1.70) | 0.542 | 2.01 (1.06,3.83) | 0.034 | 0.79 (0.37,1.70) | 0.542 |
| **Condomless sex after using drugs in the past year** |  |  |  |  |  |  |  |  |
| No | Ref |  | Ref |  | Ref |  | Ref |  |
| Yes | 3.77 (2.53,5.61) | <0.001 | 1.14 (0.63,2.06) | 0.656 | 3.77 (2.53,5.61) | <0.001 | 1.14 (0.63,2.06) | 0.656 |
| **Condomless sex with multiple partners after using drugs in the past year** |  |  |  |  |  |  |  |  |
| No | Ref |  | Ref |  | Ref |  | Ref |  |
| Yes | 5.15 (2.95,9.00) | <0.001 | 2.37 (1.18,4.75) | 0.016 | 5.15 (2.95,9.00) | <0.001 | 2.37 (1.18,4.75) | 0.016 |
| *Note*: CI=confidence interval; CNY=Chinese Yuan (1 CNY=0.1412 USD)  NA=Not applicable, which indicated the P-value of the particular variable in univariate analysis was ≥0.05  ^a^ Consistent parameters in the “Frequent testing vs. Infrequent testing” and “Ever testing vs. No testing” indicated that the effect of the particular variable was symmetrical across categories of HIV testing frequency, which could be regarded that proportional odds assumption was not violated.  ^b^ The variable “AIDS knowledge score” was calculated based on responses to eight yes-or-no-or-unclear statements regarding HIV infection and prevention adopted from China national sentinel surveillance questionnaires, which were adapted and tailored for different populations. Each correct answer scored one point with a maximum of eight points. The total points were scaled into two units of analysis: less than six correct answers and six or more correct answers. | | | | | | | | |

**Table S10. Results of univariate and multivariate PPOMs of MCSW**

|  | **Ever testing vs. (No testing)** | | | | **Frequent testing vs. (Infrequent testing) ^a^** | | | |
| --- | --- | --- | --- | --- | --- | --- | --- | --- |
| **Variables** | **OR (95%CI)** | **P-value** | **AOR (95%CI)** | **P-value** | **OR (95%CI)** | **P-value** | **AOR (95%CI)** | **P-value** |
| **Age (years)** |  |  |  |  |  |  |  |  |
| 18-30 | Ref |  |  |  | Ref |  |  |  |
| 31-40 | 0.69 (0.44,1.09) | 0.114 |  |  | 0.69 (0.44,1.09) | 0.114 |  |  |
| >40 | 0.94 (0.48,1.85) | 0.856 |  |  | 0.94 (0.48,1.85) | 0.856 |  |  |
| **Ethnicity** |  |  |  |  |  |  |  |  |
| Han | Ref |  |  |  | Ref |  |  |  |
| Other | 0.49 (0.10,2.48) | 0.389 |  |  | 0.49 (0.10,2.48) | 0.389 |  |  |
| **Local household** |  |  |  |  |  |  |  |  |
| Yes | Ref |  | Ref |  | Ref |  | Ref |  |
| No | 1.66 (1.10,2.50) | 0.015 | 1.46 (0.95,2.24) | 0.084 | 1.66 (1.10,2.50) | 0.015 | 1.46 (0.95,2.24) | 0.084 |
| **Local living time** |  |  |  |  |  |  |  |  |
| ≤ 2 years | Ref |  |  |  | Ref |  |  |  |
| > 2 years | 0.95 (0.59,1.54) | 0.837 |  |  | 0.95 (0.59,1.54) | 0.837 |  |  |
| **Education level** |  |  |  |  |  |  |  |  |
| Senior high school and lower | Ref |  | Ref |  | Ref |  | Ref |  |
| College and above | 1.51 (0.99,2.30) | 0.056 | 1.36 (0.85,2.15) | 0.198 | 1.51 (0.99,2.30) | 0.056 | 1.36 (0.85,2.15) | 0.198 |
| **Monthly income (CNY)** |  |  |  |  |  |  |  |  |
| ≤ 3000 | Ref |  |  |  | Ref |  |  |  |
| 3001-5000 | 1.04 (0.60,1.80) | 0.883 |  |  | 1.04 (0.60,1.80) | 0.883 |  |  |
| > 5000 | 1.22 (0.74,2.02) | 0.439 |  |  | 1.22 (0.74,2.02) | 0.439 |  |  |
| **Marital status** |  |  |  |  |  |  |  |  |
| Unmarried/divorced/ widowed | Ref |  | Ref |  | Ref |  | Ref |  |
| Married/cohabitating | 0.67 (0.45,1.01) | 0.053 | 0.69 (0.45,1.08) | 0.102 | 0.67 (0.45,1.01) | 0.053 | 0.69 (0.45,1.08) | 0.102 |
| **AIDS knowledge score ^b^** |  |  |  |  |  |  |  |  |
| < 6 | Ref |  | Ref |  | Ref |  | Ref |  |
| ≥ 6 | 2.17 (1.15,4.08) | 0.016 | 1.92 (0.99,3.75) | 0.055 | 2.17 (1.15,4.08) | 0.016 | 1.92 (0.99,3.75) | 0.055 |
| **Alcohol consumption in the past 3 months** |  |  |  |  |  |  |  |  |
| Never or occasionally | Ref |  | Ref |  | Ref |  | Ref |  |
| 1-4 times a month | 0.51 (0.31,0.82) | 0.005 | 0.53 (0.32,0.88) | 0.014 | 0.51 (0.31,0.82) | 0.005 | 0.53 (0.32,0.88) | 0.014 |
| ≥ once a week | 0.51 (0.31,0.85) | 0.010 | 0.64 (0.37,1.09) | 0.101 | 0.51 (0.31,0.85) | 0.010 | 0.64 (0.37,1.09) | 0.101 |
| **Condomless sex with female sex worker/ non-commercial temporary partner in past year** |  |  |  |  |  |  |  |  |
| No | Ref |  |  |  | Ref |  |  |  |
| Yes | 0.73 (0.48,1.10) | 0.130 |  |  | 0.73 (0.48,1.10) | 0.130 |  |  |
| **Number of female sex workers/non-commercial temporary partners in past year** |  |  |  |  |  |  |  |  |
| < 2 | Ref |  | Ref |  | Ref |  | Ref |  |
| ≥ 2 | 0.66 (0.43,1.00) | 0.052 | 0.68 (0.44,1.06) | 0.090 | 0.66 (0.43,1.00) | 0.052 | 0.68 (0.44,1.06) | 0.090 |
| *Note*: CI=confidence interval; CNY=Chinese Yuan (1 CNY=0.1412 USD)  NA=Not applicable, which indicated the P-value of the particular variable in univariate analysis was ≥0.05  ^a^ Consistent parameters in the “Frequent testing vs. Infrequent testing” and “Ever testing vs. No testing” indicated that the effect of the particular variable was symmetrical across categories of HIV testing frequency, which could be regarded that proportional odds assumption was not violated.  ^b^ The variable “AIDS knowledge score” was calculated based on responses to eight yes-or-no-or-unclear statements regarding HIV infection and prevention adopted from China national sentinel surveillance questionnaires, which were adapted and tailored for different populations. Each correct answer scored one point with a maximum of eight points. The total points were scaled into two units of analysis: less than six correct answers and six or more correct answers. | | | | | | | | |

**Table S11. Results of univariate and multivariate PPOMs of SNP**

|  | **Ever testing vs. (No testing)** | | | | **Frequent testing vs. (Infrequent testing) ^a^** | | | |
| --- | --- | --- | --- | --- | --- | --- | --- | --- |
| **Variables** | **OR (95%CI)** | **P-value** | **AOR (95%CI)** | **P-value** | **OR (95%CI)** | **P-value** | **AOR (95%CI)** | **P-value** |
| **Age (years)** |  |  |  |  |  |  |  |  |
| 18-30 | Ref |  |  |  | Ref |  |  |  |
| 31-40 | 1.28 (0.67,2.46) | 0.452 |  |  | 1.28 (0.67,2.46) | 0.452 |  |  |
| >40 | 1.13 (0.61,2.11) | 0.694 |  |  | 1.13 (0.61,2.11) | 0.694 |  |  |
| **Ethnicity** |  |  |  |  |  |  |  |  |
| Han | Ref |  | Ref |  | Ref |  | Ref |  |
| Other | 0.45 (0.26,0.78) | 0.005 | 0.42 (0.23,0.77) | 0.005 | 0.14 (0.08,0.26) | <0.001 | 0.18 (0.09,0.35) | <0.001 |
| **Local household** |  |  |  |  |  |  |  |  |
| Yes | Ref |  | Ref |  | Ref |  | Ref |  |
| No | 0.56 (0.32,0.98) | 0.043 | 0.55 (0.30,0.99) | 0.047 | 1.32 (0.84,2.07) | 0.224 | 1.20 (0.72,2.01) | 0.482 |
| **Local living time** |  |  |  |  |  |  |  |  |
| ≤ 2 years | Ref |  |  |  | Ref |  |  |  |
| > 2 years | 1.50 (0.76,2.96) | 0.246 |  |  | 1.50 (0.76,2.96) | 0.246 |  |  |
| **Education level** |  |  |  |  |  |  |  |  |
| Senior high school and lower | Ref |  | Ref |  | Ref |  | Ref |  |
| College and above | 1.93 (0.94,3.94) | 0.072 | 2.04 (0.89,4.68) | 0.092 | 3.76 (2.28,6.20) | <0.001 | 2.67 (1.45,4.90) | 0.002 |
| **Monthly income (CNY)** |  |  |  |  |  |  |  |  |
| ≤ 3000 | Ref |  | Ref |  | Ref |  | Ref |  |
| 3001-5000 | 2.73 (1.67,4.46) | <0.001 | 1.45 (0.81,2.60) | 0.206 | 2.73 (1.67,4.46) | <0.001 | 1.45 (0.81,2.60) | 0.206 |
| > 5000 | 0.76 (0.35,1.67) | 0.492 | 0.39 (0.15,1.00) | 0.050 | 0.76 (0.35,1.67) | 0.492 | 0.39 (0.15,1.00) | 0.050 |
| **AIDS knowledge score ^b^** |  |  |  |  |  |  |  |  |
| < 6 | Ref |  | Ref |  | Ref |  | Ref |  |
| ≥ 6 | 2.41 (0.99,5.83) | 0.052 | 1.95 (0.73,5.20) | 0.182 | 0.87 (0.39,1.95) | 0.734 | 0.64 (0.26,1.60) | 0.340 |
| **Alcohol consumption in the past 3 months** |  |  |  |  |  |  |  |  |
| Never or occasionally | Ref |  |  |  | Ref |  |  |  |
| 1-4 times a month | 1.10 (0.60,2.02) | 0.769 |  |  | 1.10 (0.60,2.02) | 0.769 |  |  |
| ≥ once a week | 1.12 (0.51,2.44) | 0.784 |  |  | 1.12 (0.51,2.44) | 0.784 |  |  |
| **Condomless sex with spouse in the past year** |  |  |  |  |  |  |  |  |
| No | Ref |  | Ref |  | Ref |  | Ref |  |
| Yes | 0.43 (0.20,0.96) | 0.040 | 0.68 (0.24,1.90) | 0.462 | 0.43 (0.20,0.96) | 0.040 | 0.68 (0.24,1.90) | 0.462 |
| **Frequency of sexual behavior with spouse in the past year** |  |  |  |  |  |  |  |  |
| Never | Ref |  | Ref |  | Ref |  | Ref |  |
| < once a week | 1.22 (0.66,2.27) | 0.524 | 1.22 (0.62,2.41) | 0.568 | 0.40 (0.24,0.64) | <0.001 | 0.56 (0.33,0.96) | 0.035 |
| ≥ once a week | 1.31 (0.67,2.56) | 0.438 | 1.25 (0.58,2.71) | 0.567 | 0.25 (0.15,0.43) | <0.001 | 0.39 (0.21,0.72) | 0.003 |
| *Note*: CI=confidence interval; CNY=Chinese Yuan (1 CNY=0.1412 USD)  NA=Not applicable, which indicated the P-value of the particular variable in univariate analysis was ≥0.05  ^a^ Consistent parameters in the “Frequent testing vs. Infrequent testing” and “Ever testing vs. No testing” indicated that the effect of the particular variable was symmetrical across categories of HIV testing frequency, which could be regarded that proportional odds assumption was not violated.  ^b^ The variable “AIDS knowledge score” was calculated based on responses to eight yes-or-no-or-unclear statements regarding HIV infection and prevention adopted from China national sentinel surveillance questionnaires, which were adapted and tailored for different populations. Each correct answer scored one point with a maximum of eight points. The total points were scaled into two units of analysis: less than six correct answers and six or more correct answers. | | | | | | | | |
